# GEF-H1 Mediated Control of NOD1 Dependent NF-κB Activation by *Shigella* Effectors

**DOI:** 10.1371/journal.ppat.1000228

**Published:** 2008-11-28

**Authors:** Atsuko Fukazawa, Carmen Alonso, Kiyotaka Kurachi, Sonal Gupta, Cammie F. Lesser, Beth Ann McCormick, Hans-Christian Reinecker

**Affiliations:** 1 Department of Medicine, Gastrointestinal Unit and Center for the Study of Inflammatory Bowel Disease, Massachusetts General Hospital and Harvard Medical School, Boston, Massachusetts, United States of America; 2 Department of Microbiology and Molecular Genetics and Department of Medicine, Division of Infectious Diseases, Massachusetts General Hospital and Harvard Medical School, Boston, Massachusetts, United States of America; 3 Department of Pediatric Gastroenterology and Nutrition, Massachusetts General Hospital and Harvard Medical School, Boston, Massachusetts, United States of America; University of Toronto, Canada

## Abstract

*Shigella flexneri* has evolved the ability to modify host cell function with intracellular active effectors to overcome the intestinal barrier. The detection of these microbial effectors and the initiation of innate immune responses are critical for rapid mucosal defense activation. The guanine nucleotide exchange factor H1 (GEF-H1) mediates RhoA activation required for cell invasion by the enteroinvasive pathogen *Shigella flexneri*. Surprisingly, GEF-H1 is requisite for NF-κB activation in response to *Shigella* infection. GEF-H1 interacts with NOD1 and is required for RIP2 dependent NF-κB activation by H-Ala-D-γGlu-DAP (γTriDAP). GEF-H1 is essential for NF-κB activation by the *Shigella* effectors IpgB2 and OspB, which were found to signal in a NOD1 and RhoA Kinase (ROCK) dependent manner. Our results demonstrate that GEF-H1 is a critical component of cellular defenses forming an intracellular sensing system with NOD1 for the detection of microbial effectors during cell invasion by pathogens.

## Author Summary

*Shigella* is a bacterium that causes food poisoning and serious intestinal infections with diarrheal illness. Pathogens like *Shigella* utilize intracellular active effectors to overcome the intestinal barrier and invade the host. We demonstrate that intestinal epithelial cells can sense the disturbance of the tight junctional seal, which normally prevents access of microbes to the circulation. A signaling molecule, which is required for cell invasion by *Shigella*, also activates messengers that activate immune defenses. This pathway of intestinal pathogen detection is activated by *Shigella* products, which are injected into host cells by the pathogen and depends on intracellular microbial recognition receptors. The detection of altered cellular function by bacterial effectors may be important for the ability to rapidly respond to barrier disruption in the intestine with the attraction and activation of immune cells to defend against the intruders.

## Introduction

The tight junctions (TJs) of the intestinal epithelium act as a defensive barrier against microbial invaders and many pathogenic bacteria have developed mechanisms to overcome the tight junctional seal to exploit the intestinal epithelium as a replicative niche or to allow entry and dissemination into the host. TJs are multiprotein complexes which consist of transmembrane components, scaffolding proteins and signaling molecules that have the potential to initiate host immune surveillance upon disruption of the tight junctional seal [1]–[4]. GEF-H1 was originally identified in mice as an oncoprotein member of the DBL family that activates RhoA in hematopoietic cells [5],[6]. GEF-H1 can associate with microtubules in non-polarized epithelial cells or the actin cytoskeleton in polarized epithelial cells and has been proposed to mediate cross talk between the two filament types [5]–[8]. GEF-H1 associates with cingulin within TJs of epithelial cells and regulates paracellular permeability [6]–[9].

*Shigella* species are human pathogens capable of colonizing the intestinal epithelium by exploiting epithelial cell functions and circumventing the host innate immune response [10]. The enteroinvasive pathogen *S. flexneri* can specifically target TJs to overcome the intestinal barrier and gain access to the basolateral membrane compartments of intestinal epithelial cells [11], a prerequisite for the invasion of epithelial cells [10]. *Shigella* cell invasion depends on the release of a subset of effectors through the type III secretion system (T3SS) both around the bacterial surface and directly into the host cell [12],[13]. It is known that the ability of *Shigella* to invade epithelial cells and subsequently spread from cell to cell is pivotal in establishing intestinal infection. This process is associated with a strong inflammatory response, but the bacterial effectors and host cell response mechanisms leading to defense activation are not well understood. [12],[14].

Signaling through nucleotide binding and oligomerization domain (NOD)-like receptor (NLR) NOD1 provides the intestinal epithelium with a mechanism for activating innate immunity during infection by invasive pathogenic Gram negative bacteria [15],[16]. Intracellular pattern recognition receptors such as NOD1 can detect the D-Glu-meso-diaminopimelic acid (DAP) dipeptide of *S. flexneri* in macrophages and activate NF-κB [17],[18]. *S. flexneri* employs a diverse array of effectors to alter the host actin cytoskeleton and innate immune responses including regulators for Rho GTPases and NF-κB [10], [19]–[24]. However, it has not been established which bacterial and host mediators signal the disruption of the apical junctional complex to initiate cellular defense responses in the intestinal epithelium.

In this study, we demonstrate that GEF-H1 is a central component of pathogen recognition by NOD1. GEF-H1 and NOD1 together not only detect the presence of peptidoglycan (PGN)-derived muropeptides but also signal in response to *Shigella* effectors in the cytoplasm. GEF-H1 is recruited into bacterial invasion sites of *S. flexneri* and the subsequent RhoA activation is required for cell invasion. In addition, GEF-H1 is requisite for the activation of NF-κB dependent gene expression during *Shigella* cell invasion. NF-κB activation by GEF-H1 is independent from the detection of bacterial products by Toll-like receptor (TLR) and cytokine receptor signaling. Instead, GEF-H1 interacts with NOD1 and is required for NF-κB activation in response to γTriDAP. Importantly, we find that the *Shigella* effectors IpgB2 and OspB activate NF-κB by a mechanism that depends on both NOD1 and GEF-H1 and requires ROCK activation. GEF-H1 is a central component in a detection system that directs NF-κB activation in RhoA and RIP2 dependent pathways initiated by the action of bacterial effectors and intracellular pathogen pattern recognition.

## Results

### GEF-H1 is recruited into membrane ruffles induced by *S. flexneri* at tight junctions of polarized epithelial cells

**Figure 1:**
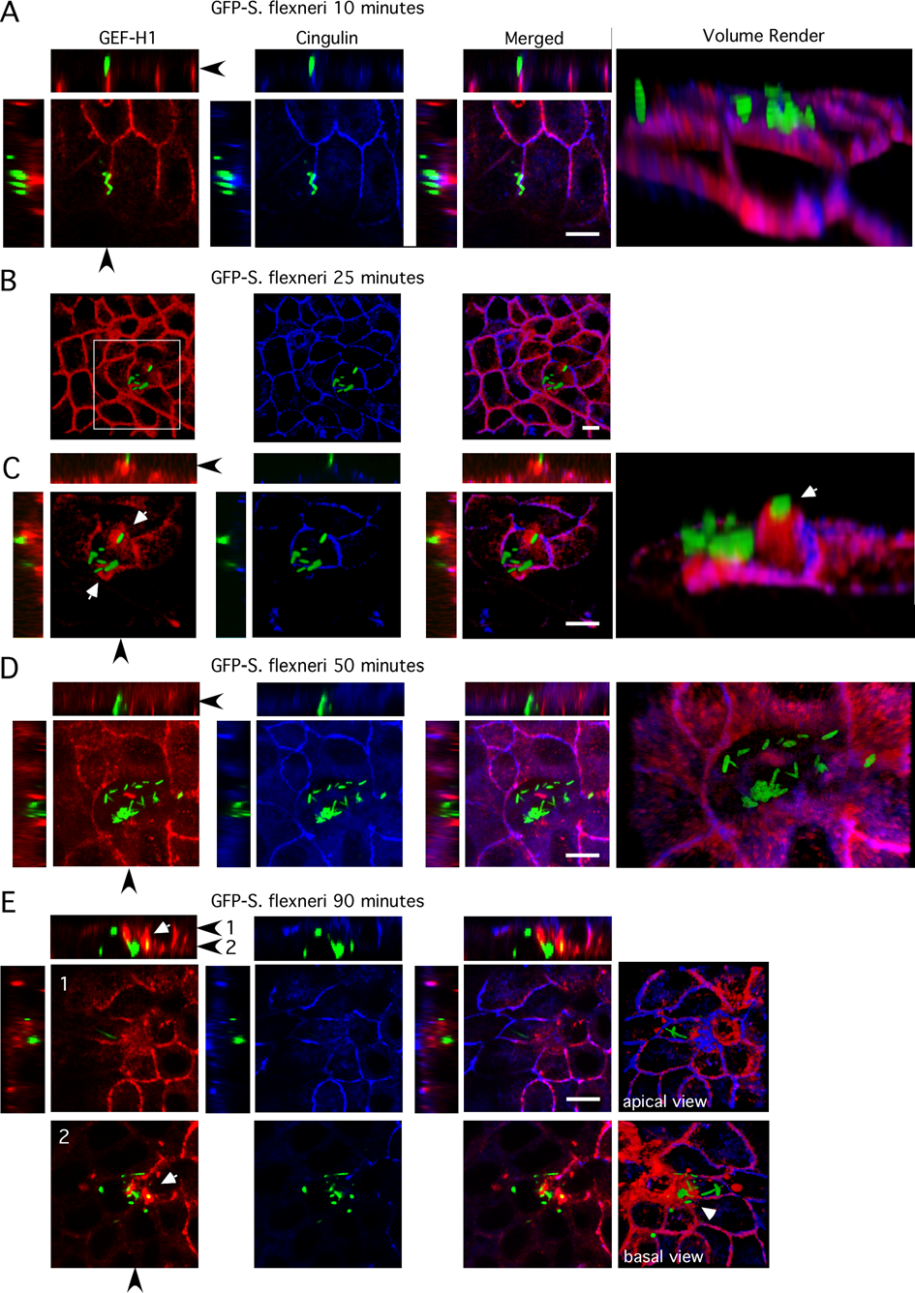
GEF-H1 is recruited into membrane ruffles induced by *S. flexneri*. XY, XZ and YZ sections as well as three-dimensional reconstructions (volume render) of confocal image series of GEF-H1 recruitment to the invasion sites of *S. flexneri*. Polarized MDCK monolayers were exposed to green fluorescent protein (GFP)-expressing *S. flexneri* and then fixed and stained at different time points. Endogenous GEF-H1 was stained with anti-GEF-H1 antibody and Texas red secondary antibody. Cingulin was stained with anti-cingulin antibody and Cy5 secondary antibody. Black arrows locate the cross section level. Bars indicate 5 µm. (A) Initially, *S. flexneri* was found attached to TJs, co-localizing with GEF-H1 and cingulin. (B and C) Thereafter, *Shigella* gained access to the TJs and induced membrane ruffles and pedestals that extended above the TJs. White arrows represent area indicated by white frame in (B). GEF-H1 was recruited from tight junctional complexes into bacterial entry sites, while cingulin was not and remained associated with the TJs. (D) Subsequently, *Shigella* was found within the cytoplasm of MDCK cells and GEF-H1 and cingulin were removed from the tight junctional area of infected cells. (E) Upon cell invasion, *Shigella* was found inside the epithelial monolayer either free in the cytoplasm or associated with intracellular vesicles containing GEF-H1 (white arrows). The cytoplasm of the infected cells appeared to retract and the neighboring epithelial cells started to close above the infected cells.

Interaction of *S. flexneri* with the polarized epithelium results in the redistribution of TJ associated proteins which results in the loss of barrier function and allows access to the basolateral membrane compartment to facilitate cell invasion [11]. We utilized GFP expressing *S. flexneri* to determine the subcellular redistribution of GEF-H1 and its binding partner cingulin in TJs [7] during invasion of polarized Madden Darby canine kidney (MDCK) model epithelial cell monolayers. As demonstrated in **Figure 1A**, *S. flexneri* initially was found specifically attached to cell-cell contacts and co-localized with GEF-H1 and cingulin in TJs of polarized MDCK cell monolayers. Subsequently, *Shigella* gained access to the tight junctional structure and altered its composition. As demonstrated in **Figure 1B and C**, *Shigella* induced membrane ruffles that extended above the TJs in MDCK cells. GEF-H1 but not cingulin was found to be recruited into bacterial entry sites (**Figure 1B and C**). When *Shigella* began to gain access to the cytoplasm, both GEF-H1 and cingulin were removed from the tight junctional area of infected cells (**Figure 1D**). Afterwards, *Shigella* was found either free in the cytoplasm or in compartments which were GEF-H1 positive and the neighboring epithelial cells started to contract above the infected cell (**Figure 1E**). Of note, cells in close proximity to infected cells demonstrated increased GEF-H1 expression in the cytoplasm. Together, these data demonstrate that GEF-H1 is recruited into membrane ruffles induced upon disruption of TJs and the modifications of the cytoskeleton by *Shigella*.

### GEF-H1 function is required for host cell invasion by *S. flexneri*

**Figure 2:**
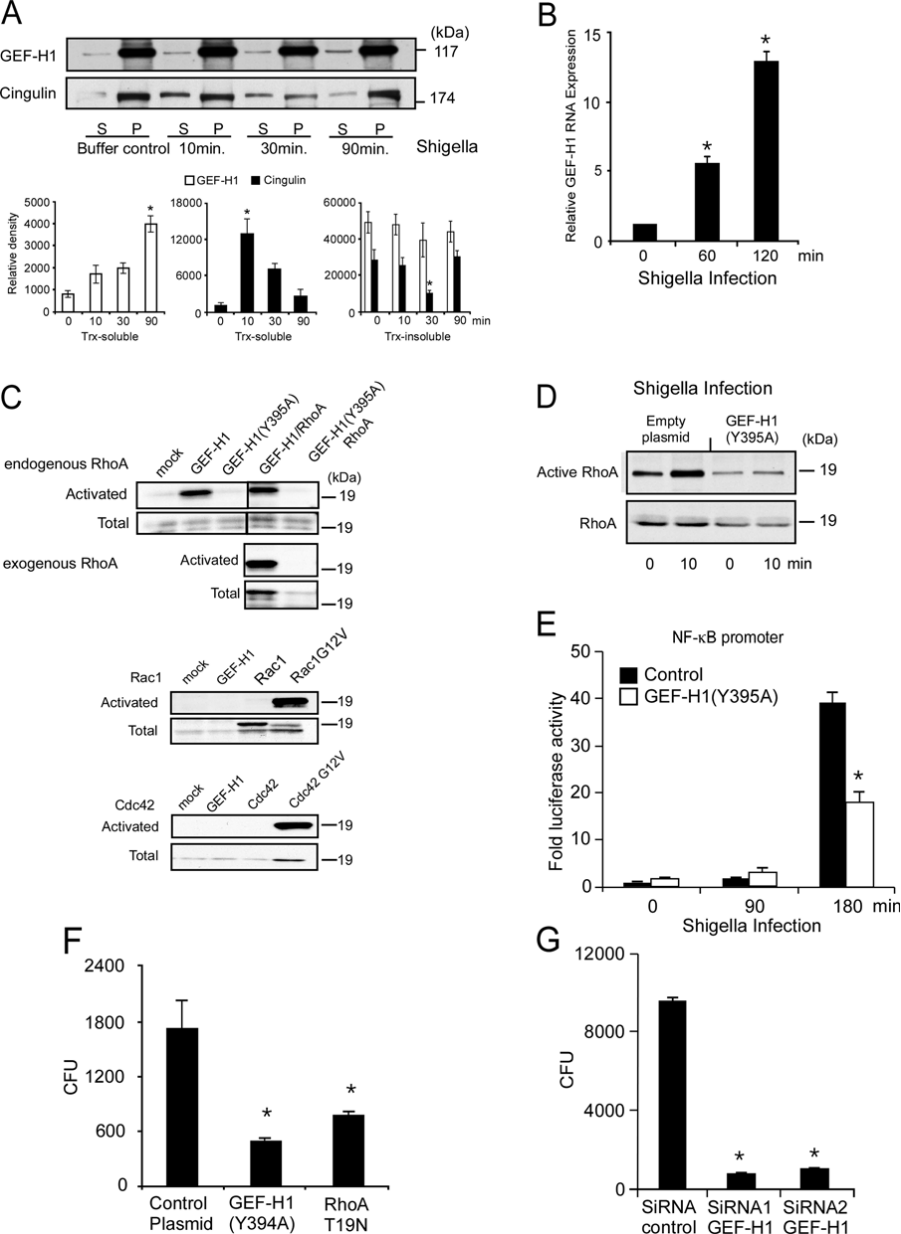
GEF-H1 mediates RhoA activation during *S. flexneri* cell invasion. (A) Western blot analysis of subcellular distribution of GEF-H1 and cingulin upon *S. flexneri* invasion of MDCK cell monolayer. Densitometric analysis of GEF-H1 and cingulin in soluble and insoluble protein fractions during *Shigella* infection. Bars represent mean±SD (* p<0.01 compared to the rest of time points). (B) Real-time PCR assessment of GEF-H1 expression in response to *S. flexneri* invasion of MDCK cells. Bars represent mean±SD (* p<0.01 compared to non-infected cells). (C) GST pull down assays to assess activation of small GTPases in response to GEF-H1 expression. HEK293 cells were transfected with GEF-H1, RhoA, Rac1 and Cdc42 expression vectors, GEF-H1 (Y395A) mutant and constitutive active mutants for Rac1 (G12V) and Cdc42 (G12V). (D) GST pull down assays to assess activation of RhoA to *S. flexneri* infection. HEK293 cells were transfected with GEF-H1 mutant (Y395A) defective in nucleotide exchange or the empty plasmid as a control and incubated with *S. flexneri* for 10 minutes. Expression of GEF-H1 (Y395A) decreased baseline activation and prevented the rapid activation of RhoA during *Shigella* invasion of HEK293 cells. (E) NF-κB activation in response to *S. flexneri* invasion of HEK293 cells in the absence or presence of dominant negative GEF-H1 (Y395A) mutant. Bars represent mean±SD (* p<0.05 compared to control). (F) Gentamicin protection assays of *S. flexneri* infection of HEK293 cells transfected with GEF-H1 (Y395A) or dominant negative RhoA (T19N) expression vectors. Bars represent mean±SD (* p<0.05 compared to control plasmid). (G) Gentamicin protection assays of *S. flexneri* infection of HEK293 cells in the presence of two different GEF-H1 siRNAs. Bars represent mean±SD (* p<0.05 compared to control siRNA).

The recruitment of GEF-H1 to invasion sites raised the possibility that GEF-H1 was targeted by bacterial effectors to activate Rho GTPases at the basolateral membrane compartments as a prerequisite for cell invasion by *S. flexneri* [25]. We therefore determined the requirement of GEF-H1 and its contribution to Rho GTPase activation during *Shigella* cell invasion. Invasion of MDCK monolayers by *S. flexneri* was associated with an increase of GEF-H1 expression in Triton X-soluble protein fractions. The membrane associated pool of GEF-H1 remained high during the interaction of *S. flexneri* with the polarized epithelium, suggesting that GEF-H1 was rapidly redistributed from apical junctions to new membrane associated binding partners at bacterial entry sites or basolateral membrane compartments (**Figure 2A**). Cingulin was redistributed from the TJ associated Triton X-insoluble to the -soluble protein fractions during the initial phase of *Shigella* cell invasion potentially initiating the release of GEF-H1 (**Figure 2A**). However, within 90 minutes, cingulin levels in TJ associated membrane fractions recovered. In contrast, cytosolic GEF-H1 protein levels continued to increase over the 90 minute observation period (**Figure 2A**). The increase in cytosolic GEF-H1 expression observed in soluble protein fractions may not only result from subcellular redistribution, but could also include protein neosynthesis, since GEF-H1 mRNA expression was upregulated 13.3±0.5-fold within 2 hours of *S. flexneri* infection in MDCK cells (**Figure 2B**).

Increases in expression of GEF-H1 in HEK293 cells led to the activation of RhoA, but not Cdc42 and Rac1, as demonstrated by the induction of Rhotekin binding to activated RhoA but not of PAK1 to Rac1 or Cdc42 in GST pull down assays (**Figure 2C**). GEF-H1 was responsible for RhoA activation during cell invasion, since expression of a GEF-H1 mutant (Y395A) defective in nucleotide exchange prevented the activation of RhoA during *Shigella* invasion of HEK293 cells (**Figure 2D**). In addition, the expression of the catalytically inactive GEF-H1 reduced the basal levels of RhoA activation (**Figure 2D**). Furthermore, expression of GEF-H1 (Y395A) also reduced NF-κB reporter gene activation by 52±5% during infection of HEK293 cells by *S. flexneri* (**Figure 2E**). Expression of GEF-H1 (Y395A) or RhoA (T19N) (**Figure 2F**) or the depletion of GEF-H1 expression by two distinct siRNAs significantly reduced cell invasion of HEK293 by *S. flexneri* (**Figure 2G**). Together these findings demonstrate a critical function of GEF-H1 in RhoA activation required for cell invasion by *S. flexneri*.

### GEF-H1 mediates NF-κB activation during *Shigella* cell invasion

**Figure 3:**
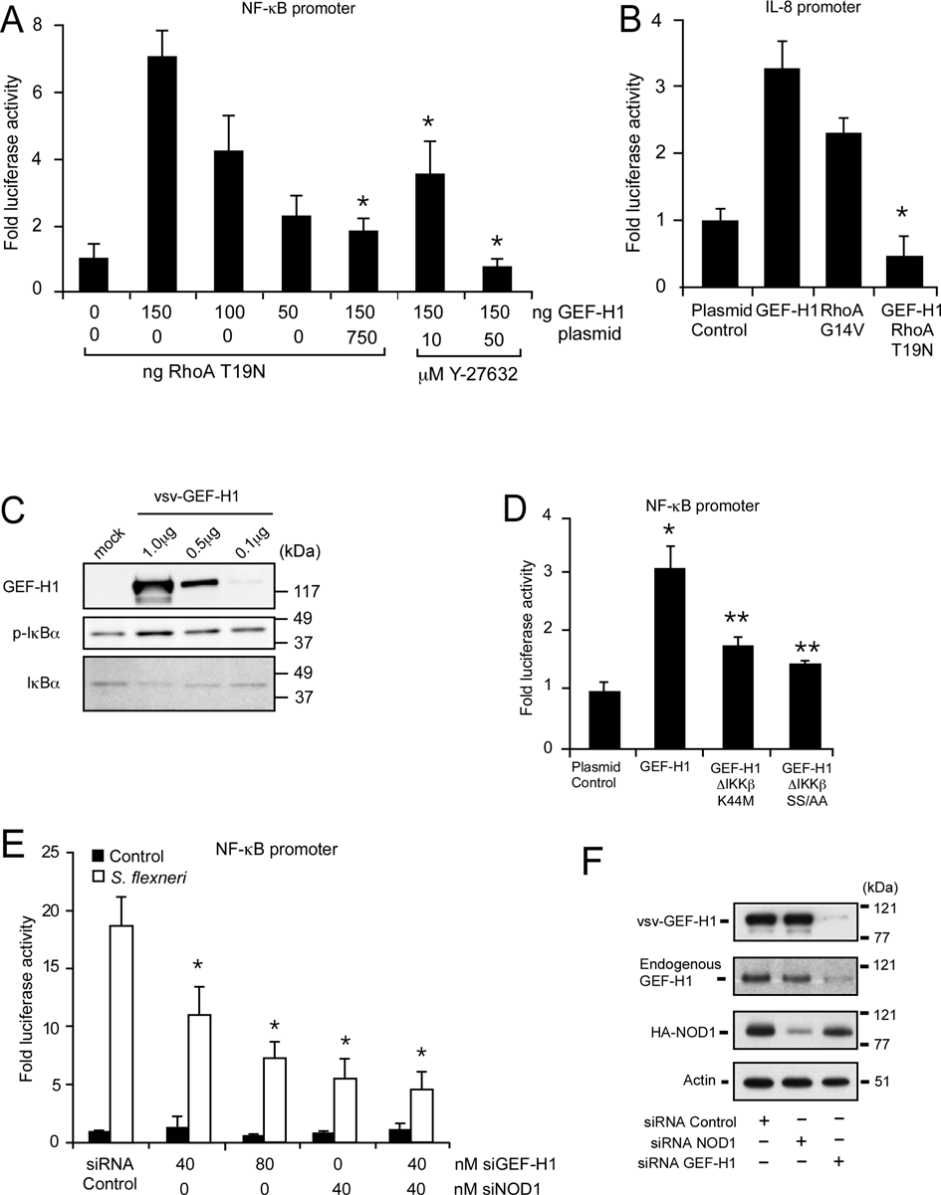
GEF-H1 mediates RhoA dependent NF-κB activation. (A) GEF-H1 mediated NF-κB reporter gene expression in the absence or presence of dominant negative RhoA (T19N) or the ROCK inhibitor Y27632 in HEK293. Bars represent mean±SD (*p<0.05 compared to GEF-H1 alone). (B) GEF-H1 and RhoA mediated IL-8 reporter gene expression. HEK293 cells were transfected with GEF-H1 in the absence or presence of dominant negative RhoA (T19N) or the constitutive active RhoA mutant (G14V) plasmids. Bars represent mean±SD (*p<0.05, compared to GEF-H1 alone). (C) Western blot analysis of IκBα phosphorylation and protein degradation in response to GEF-H1 expression in HEK293 cells. (D) NF-κB reporter activation by GEF-H1 in the absence or presence of IKKβ kinase negative mutant K44M or dominant negative SS/AA mutant in HEK293 cells. Bars represent mean±SD (*p<0.05 compared to control, **p<0.05 compared to GEF-H1 alone). (E) NF-κB reporter activation by *S. flexneri* in HEK293 cells after depletion of NOD1 and GEF-H1 with specific siRNAs. Bars represent mean±SD (* p<0.05 compared to Shigella responses in the presence of control siRNAs). (F) Western blot demonstrating that specific and effective knock down of endogenous and exogenous GEF-H1 as well as overexpressed NOD1 in HEK293 cells.

Since RhoA activation has been linked to the activation of NF-κB [26], we determined whether enhanced cytoplasmic GEF-H1 expression was linked to NF-κB activation during *S. flexneri* cell invasion. Expression of GEF-H1 in HEK293 cells induced activation of NF-κB in a dose dependent fashion (**Figure 3A**). The activation of the NF-κB pathway by GEF-H1 was dependent on its RhoA specific GEF activity. Co-expression of the dominant negative allele of RhoA (T19N) prevented GEF-H1 mediated NF-κB activation in HEK293 cells (**Figure 3A**). Furthermore, the RhoA target kinase p160/ROCK was required for GEF-H1 mediated NF-κB activation, since the presence of ROCK inhibitor Y27632 decreased GEF-H1 mediated NF-κB activation (**Figure 3A**). GEF-H1 expression induced IL-8 promoter activity in HEK293 cells which was prevented in the presence of the RhoA (T19N) mutant (**Figure 3B**). GEF-H1 overexpression resulted in the phosphorylation and subsequent degradation of IκBα (**Figure 3C**). Furthermore, the activation of NF-κB through GEF-H1 was partially dependent on the inhibitor of NF-κB kinase complex subunit β (IKKβ), since either the kinase negative mutant IKKβ (K44M) or the dominant negative mutant IKKβ (SS/AA) inhibited GEF-H1 mediated activation of NF-κB (**Figure 3D**).

NF-κB activation upon *S. flexneri* cell invasion has been linked to recognition of this intracellular pathogen by NOD1 [17]. Consistent with these observations, depletion of NOD1 with specific siRNAs but not control siRNAs inhibited *S. flexneri* mediated NF-κB activation in HEK293 cells by 66±8% (**Figure 3E**). However, NF-κB activation in response to infection by *S. flexneri* was also inhibited in a dose dependent manner by the siRNA mediated depletion of GEF-H1 (**Figure 3E**). Inhibition of GEF-H1 and NOD1 expression together decreased NF-κB activation upon *S. flexneri* infection by 78±12% (**Figure 3E**). Depletion of either GEF-H1 or NOD1 by the utilized siRNAs was specific (**Figure 3F**). Together, these data demonstrate that GEF-H1 is required for NF-κB activation during cell invasion by *S. flexneri* and that GEF-H1 induced RhoA activation can contribute to the activation of the canonical NF-κB pathway and induction of IL-8 transcription.

### GEF-H1 is required for NOD1 dependent NF-κB activation

**Figure 4:**
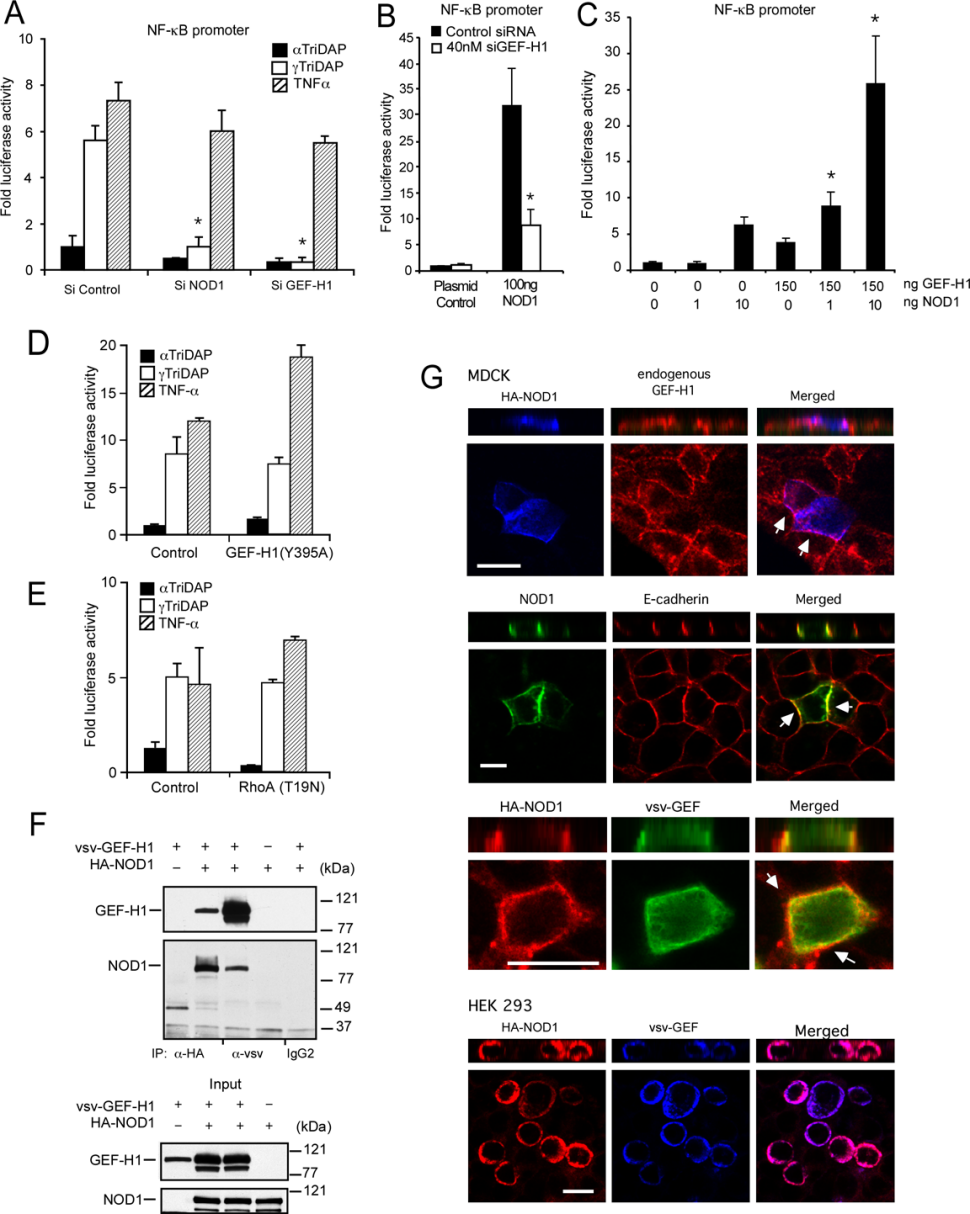
GEF-H1 interacts with NOD1 and is required for NOD1 dependent NF-κB activation. (A) NF-κB activation in response to active and inactive NOD1 ligands in the absence or presence of GEF-H1 and NOD1 siRNA in HEK293 cells. Bars represent mean±SD (* p<0.01, compared to responses in the presence of control siRNA and γTriDAP). (B) NF-κB activation in response to overexpression of NOD1 in the presence of control or GEF-H1 siRNA in HEK293 cells. Bars represent mean±SD (* p<0.01 compared to control siRNA). (C) NF-κB activation in response to transfection of indicated amounts of expression vectors encoding GEF-H1 and NOD1 in HEK293 cells. Bars represent mean±SD (*p<0.01 compared to GEF-H1 and NOD1 alone). (D and E) RhoA activation is not required for γTriDAP and TNFα signaling. NF-κB activation in response to active and inactive NOD1 ligands and TNFα in HEK293 cells transfected with GEF-H1 (Y395A), dominant negative RhoA (T19N) or control expression vectors. Bars represent mean±SD (*p = NS, control vector vs GEF-H1 (Y395) or RhoA (T19) in the presence of γTriDAP). (F) Co-immunoprecipitation of GEF-H1 and NOD1 with indicated antibodies in HEK293 cells. (G) Confocal microscopic image analysis of the subcellular localization of NOD1, GEFH1 and E-cadherin. MDCK monolayers and HEK 293 cells were transfected with HA-NOD1 vector alone or in addition to vsv-GEF-H1 and then fixed and stained for confocal microscopic analysis. Endogenous GEF-H1 was stained with anti-GEF-H1 antibody and Texas red secondary antibody. E-cadherin was stained with anti-E-cadherin antibody and Texas red secondary antibody. HA-NOD1 was stained with anti-HA antibody and Cy5, Texas Red or FITC secondary antibody; vsv-GEF-H1 was stained with anti-vsv antibody and Cy5 or FITC secondary antibody. Bars indicate 5 µm.

We next determined the role of GEF-H1 in the activation of NF-κB by the NOD1 ligand γTriDAP. In these experiments, HEK293 cells transfected with the NF-κB luciferase reporter plus NOD1, GEF-H1 or control siRNAs were exposed to either γTriDAP or control compound αTriDAP (Ala-αGlu-meso-DAP). Addition of γTriDAP to HEK293 cells induced a 5.6±0.7-fold increase in NF-κB promoter activity (**Figure 4A**). As demonstrated in **Figure 4A**, depletion of GEF-H1 prevented NF-κB activation through specific NOD1 ligands more efficiently than the inhibition of NOD1 expression (93±1.3% versus 79±7.1% reduction), implicating GEF-H1 as a critical component in the recognition of specific PGN-derived muropeptides. In contrast, depletion of neither GEF-H1 nor NOD1 significantly inhibited NF-κB activation in response to TNF-α receptor signal transduction in HEK293 cells (**Figure 4A**). Depletion of GEF-H1 also prevented NF-κB activation induced by overexpression of NOD1 by 75±5% (**Figure 4B**). Conversely, overexpression of GEF-H1 together with NOD1 synergistically upregulated NF-κB dependent gene transcription by up to 26±7-fold, while expression of GEF-H1 or NOD1 alone increased NF-κB dependent transcription by 4±0.6-fold and 3.5±0.4-fold, respectively (**Figure 4C**). However, RhoA activation was not required for signaling of the NOD1 ligand γTriDAP or TNF-α since expression of GEF-H1 (Y395A) or RhoA (T19N) failed to inhibit NF-κB activation (**Figure 4D and E**). Instead, GEF-H1 was able to interact with NOD1 directly or through intermediates, since both were found to co-immunoprecipitate when antibodies directed against either NOD1 or GEF-H1 but not control antibodies were used (**Figure 4F**). Confocal microscopic analysis revealed that NOD1 was recruited to the basolateral membrane compartment in polarized epithelial cells but co-localized with endogenous or exogenously expressed GEF-H1 in cell-cell contacts in MDCK cells or HEK-293 cells (**Figure 4G**). We concluded from these experiments that GEF-H1 is requisite for signal transduction by NOD1 independent of its GEF activity.

### GEF-H1 mediates NF-κB activation initiated by *Shigella* effectors

**Figure 5:**
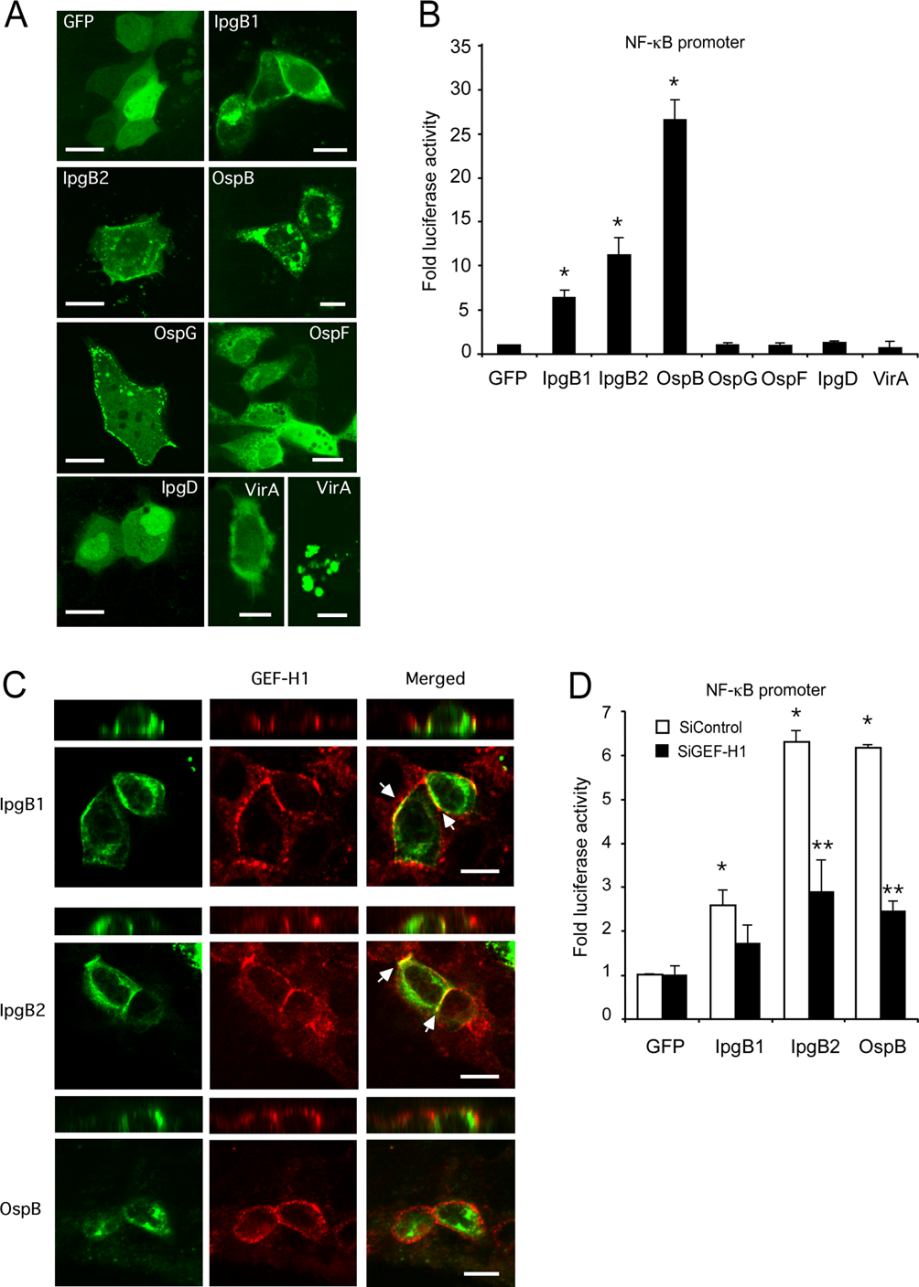
GEF-H1 and NOD1 mediate NF-κB activation induced by *S. flexneri* effectors. (A) Confocal microscopic image analysis of MDCK cells transfected with indicated GFP-tagged *S. flexneri* effectors. Bars indicate 10 µm. (B) NF-κB activation in response to GFP-tagged *S. flexneri* effectors in HEK293 cells. (C) Three-dimensional reconstructions of confocal microscopic image series of MDCK cells transfected with indicated GFP-tagged *S. flexneri* effectors and immunostained for endogenously expressed GEF-H1. Bars indicate 10 µm, arrows indicate co-localization of GEF-H1 and *S. flexneri* effectors. (D) NF-κB activation in response to IpgB1, IpgB2 and OspB expression in the absence or presence of control or GEF-H1 specific siRNAs (*p<0.01 compared to the expression of GFP control alone, **p<0.01 compared to siRNA control).

During the course of infection, *S. flexneri* modulates Rho GTPase function and NF-κB activation through a number of effectors delivered directly into host cells [19],[20]. We screened for *S. flexneri* effectors that are able to target the cytoskeleton or replace NF-κB signaling during infection (**Figure 5A and B**). When GFP tagged *Shigella* effectors were transfected into MDCK cells, IpgB1 and IpgB2 localized to cellular junctions and intracellular vesicular compartments, while OspB associated only with intracellular compartments (**Figure 5A**). OspG, which has been shown to inhibit NF-κB activation, also associated with a subcellular compartment close to the cell membrane. In contrast, OspF remained cytoplasmic, while IpgD was found to be enriched in cell nuclei. Also, VirA demonstrated a cytoplasmic staining pattern, but did not activate NF-κB, although this effector rapidly induced membrane blebbing and cell death when expressed in MDCK cells (**Figure 5A**).

When expressed as GFP fusion proteins in HEK293 cells, IpgB2, OspB and, to a lesser extent, IpgB1, were able to activate NF-κB promotor activity by 11.3±2.5, 26.5±2.3 and 6.2±1.2-fold above baseline activity of GFP control plasmid. In contrast, OspG, OspF, IpgD, as well as VirA, failed to significantly upregulate NF-κB promoter activity (**Figure 5B**).

Furthermore, the subcellular distribution of IpgB1 and IpgB2 partially overlapped with GEF-H1 in cellular junctions of MDCK cells. In contrast, OspB remained in a cellular compartment which had very little overlap with the GEF-H1 containing membrane compartments in MDCK cells (**Figure 5C**).

Surprisingly, in the absence of GEF-H1, all *S. flexneri* effectors failed to induce significant NF-κB activation (**Figure 5D**). When expressed in MDCK cells, IpgB1, IpgB2 and OspB induced NF-κB activity 2.8±0.2, 6.5±0.7 and 6.2±0.1-fold, respectively, above background levels found in the presence of GFP alone (**Figure 5D**). SiRNA mediated depletion of GEF-H1 significantly reduced the NF-κB activation induced in the presence of IpgB2 and OspB (**Figure 5D**). Collectively, these experiments demonstrated that *Shigella* effectors can directly or indirectly activate NF-κB activation independently of muramyl dipeptides by a mechanism that requires the presence of GEF-H1.

### GEF-H1 and NOD1 are required for NF-κB activation by muropeptides and *Shigella* effectors

**Figure 6:**
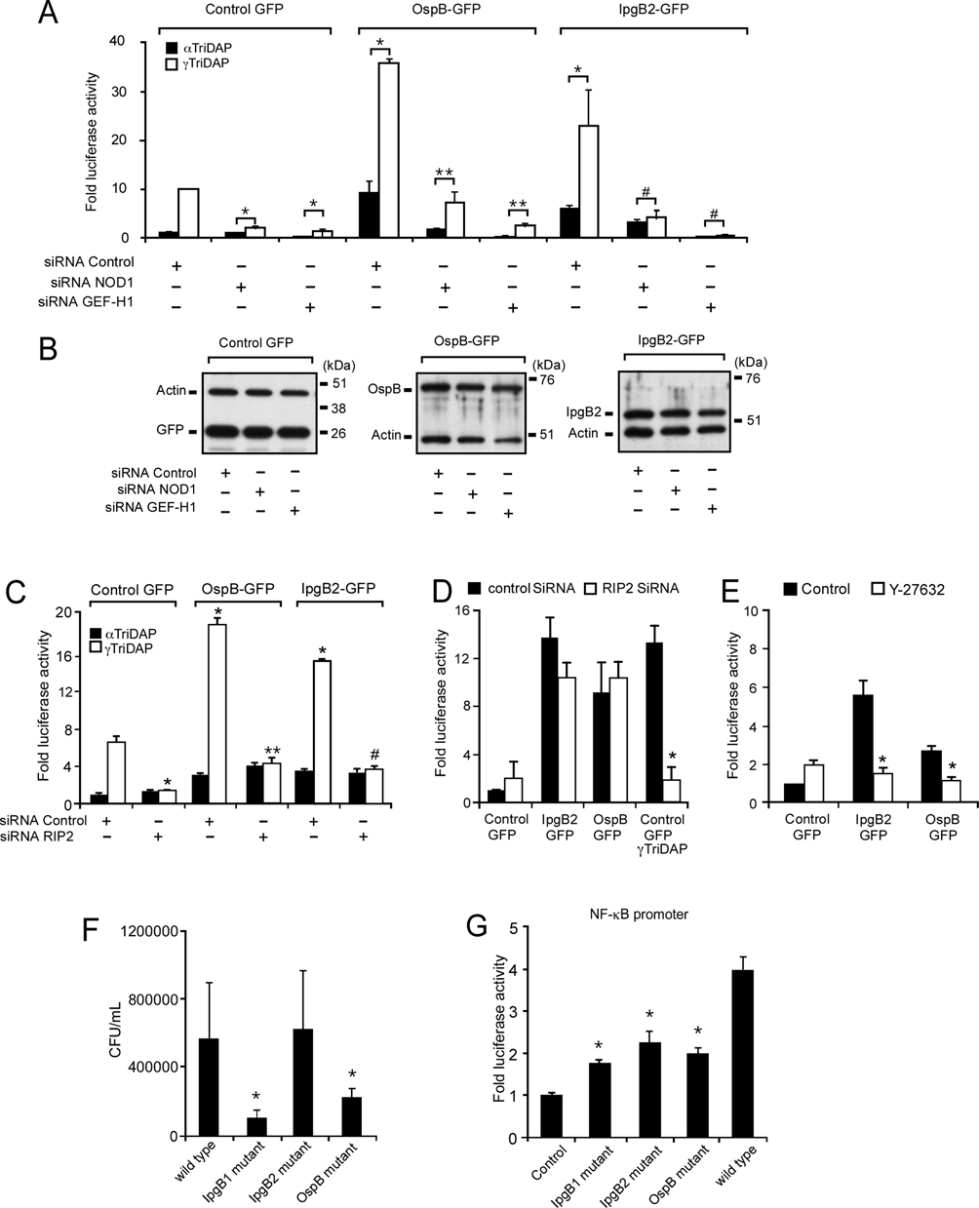
GEF-H1 and NOD1 are required for NF-κB activation by muropeptides and *Shigella* effectors. (A) NF-κB activation in response to control or active NOD1 ligand (γTriDAP) in the absence or presence of *Shigella* effectors and control, GEF-H1 or NOD1 siRNAs (* p<0.01 compared to responses in the presence of control siRNA and γTriDAP, **p<0.001 compared to responses in the presence of control siRNA and OspB, # p<0.001 compared to responses in the presence of control siRNA and IpgB2). (B) Depletion of GEF-H1 or NOD1 did not affect protein expression levels of the indicated GFP-tagged *S. flexneri* effectors in HEK293 cells. (C) NF-κB activation in response to control or active NOD1 ligand (γTriDAP) in the absence or presence of *Shigella* effectors and control or RIP2 siRNAs (*p<0.01 compared to responses in the presence of control vector, control siRNA and γTriDAP, **p<0.001 compared to responses in the presence of control siRNA, OspB and γTriDAP, #p<0.001 compared to responses in the presence of control siRNA, IpgB2 and γTriDAP). (D) IpgB2 and OspB signaling is RIP2 independent. NF-κB activation in response to control or *Shigella* effectors expression vectors in the presence of control or RIP2 siRNAs (* p<0.01 compared to responses in the presence of control siRNA). (E) IpgB2 and OspB signaling is ROCK dependent. NF-κB activation in response to control or *Shigella* effectors expression vectors in the presence or absence of ROCK inhibitor Y-27632 (*p<0.01 compared to responses in the absence of Y-27632). (F) Gentamicin protection assay with wild type and indicated *Shigella* mutants in HEK293 cells (*p<0.01 compared to wild type Shigella). (G) NF-κB promoter activation in HEK293 cells in response to infection by wild type and indicated *Shigella* mutants (*p<0.01, compared to wild type Shigella).

To further define the mechanism by which OspB and IpgB2 induces NF-κB activation, we depleted either GEF-H1 or NOD1 and expressed OspB and IpgB2 in the presence of γTriDAP or control peptide and determined NF-κB activation in HEK293 cells. Remarkably, in the presence of additional γTriDAP, NF-κB activity in OspB and IpgB2 transfected cells increased up to 35.5±0.9 and 22.7±7.5-fold, respectively, over baseline levels induced by transfection with the GFP control plasmid (**Figure 6A**). Surprisingly, the induction of NF-κB activity by γTriDAP, OspB and IpgB2 alone or in the presence of γTriDAP was dependent on NOD1, as well as GEF-H1, since depletion of both mediators by specific siRNAs but not control siRNAs reduced NF-κB activity down to baseline levels (**Figure 6A**). Depletion of GEF-H1 decreased NF-κB activity induced by γTriDAP by 87.1±5.7% by OspB in the presence of γTriDAP by 84.1±1.4% and the activity resulting from IpgB2 overexpression by 91.2±0.2% (**Figure 6A**). Depletion of NOD1 reduced the NF-κB activity induced by γTriDAP, OspB and IpgB2 by 81.2±3.3%, 72±11.4% and 82.2±5.5%, respectively (**Figure 6A**). The inhibition of NF-κB activation was more pronounced after depletion of GEF-H1 compared to NOD1, possibly due to different knockdown efficiencies of the gene specific siRNAs in these experiments. Specific siRNA mediated depletion of NOD1 or GEF-H1 were target specific and did not affect expression levels of IpgB2 or OspB in our cell system (**Figure 6B**). The synergistic increase in NF-κB activation induced by γTriDAP and *Shigella* effector together was dependent on the caspase recruitment domain (CARD)-containing serine/threonine kinase RIP2 (also known as Rick, CARDIAK, CCK and Ripk2) (**Figure 6C**). However, NF-κB activation induced by IpgB2 or OspB alone was found to be independent of RIP2 and instead was dependent on ROCK activation (**Figure 6D and E**).

We next carried out gentamicin protection assays with *Shigella* mutants deficient in IpgB1, IpgB2 or OspB to determine the role of these effectors in pathogen uptake and intracellular survival. As demonstrated in **Figure 6F**, *Shigella* deficient in IpgB2 were not impaired in their ability to invade and survive in HEK293 cells, while significantly lower numbers of *Shigella* lacking either IpgB1 or OspB were recovered from infected cells after 60 minutes compared to wild type *Shigella* (**Figure 6F**). However, *Shigella* mutants deficient in the expression of IpgB1 IpgB2 or OspB were all characterized by significantly reduced NF-κB activation during invasion of HEK293 cells compared to wild type *S. flexneri* (**Figure 6G**). These experiments established that GEF-H1 and NOD1 are central to the detection of intracellular *Shigella* effectors, in addition to their function in recognizing PGN-derived muropeptides. NF-κB activation during *Shigella* cell invasion involves both RhoA and RIP2 dependent activation pathways which are dependent on GEF-H1.

## Discussion

The epithelial interface is involved in constant cross talk with the intestinal microbiota through molecular mechanisms that integrate intestinal epithelial barrier function with mucosal immune regulation. Failure to accurately monitor the intestinal environment or respond adequately to challenges by pathogens results in a breakdown of the intestinal barrier. Multiple signaling components are localized at epithelial TJs [27],[28]. Most of these signal mediators have been identified through their function in the regulation of epithelial polarization, differentiation and growth control, but very little information exists about their contribution to mucosal host defense responses. We now show that the disruption of TJs by *Shigella* effectors is linked to the activation of innate immune responses through GEF-H1 controlling NOD1 mediated NF-κB activation.

GEF-H1 was recruited to *Shigella* induced membrane ruffles similarly to NOD1 which has been shown to be enriched in bacterial entry sites in an actin dependent mechanism also required for signal transduction [29]. *Shigella* effectors secreted by the T3SS could initiate NF-κB signaling before the PGN release from *Shigella* multiplying within epithelial cells that has been shown to activate the NOD pathway [30].

GEF-H1 is indispensable for NOD1 mediated NF-κB activation by γTriDAP, and by the *Shigella* effectors OspB and IpgB2. However, the downstream signaling events leading to NF-κB activation were specific for either γTriDAP or the *Shigella* effectors. *S. flexneri* infection induces NOD1 oligomerization via the homophilic CARD-CARD interaction allowing transient recruitment of RIP2 and IKK, which phosphorylates IκB leading to prolonged activation of NF-κB [17],[31]. In our experiments, GEF-H1 dependent activation of NF-κB by γTriDAP and its synergistic effect on *Shigella* effector signaling was RIP2 dependent. The ability of GEF-H1 to mediate γTriDAP initiated NOD1 signal transduction was independent of the GEF function of GEF-H1 and the activation of RhoA. Instead, in γTriDAP signaling, GEF-H1 directly interacted with NOD1 serving as a signaling adaptor through protein motifs which need to be defined in future experiments. In contrast, OspB and IpgB2 mediated NF-κB activation required RhoA mediated activation of ROCK but not RIP2. These findings demonstrate that GEF-H1 is a central component of RhoA and Rip2-mediated NF-kB activation during *Shigella* cell invasion.

In the early stages of *Shigella* infection, effectors delivered by the T3SS include IpgB1, IpgB2, IpgD and VirA both around the bacterial surface and directly into the host cell. While both IpgB1 and IpgB2 associated with cellular junctions, only IpgB2 was a potent inducer of the GEF-H1 dependent activation of NF-κB. Both IpgB1 and IpgB2 have been linked to Rho GTPase function. IpgB1 is presumed to have major roles in producing membrane ruffles by activating Rac-1 through ELMO and DOCK180, a Rac-1 guanine nucleotide exchange factor [32]. IpgB1 function has been linked to the activation of RhoG, resulting in downstream activation of Rac [33]. IpgB2 is an IpgB1 homolog that binds to mDia1 which facilitates actin nucleation and the Rho kinase ROCK through its GTPase binding domains [34]. Therefore, IpgB2 may mimic the activity of RhoA in our experiments resulting in ROCK dependent NF-κB activation. Invasiveness of IpgB1 or IpgB2 mutant *Shigella* has been recently assessed [35]. Consistent with our results, *Shigella* IpgB2 mutants have been found to exhibit the same invasive capacity as the wild type strain in non-polarized and polarized intestinal epithelial cells. In contrast, IpgB1 mutants were 50% less invasive in the non-polarized epithelium but slightly more invasive in the polarized epithelium [35]. Therefore, the reduced NF-κB activation in response to IpgB2 mutants was not due to reduced cell invasion, while IpgB1 and OspB play a role in the cell invasion process itself, which may contribute to the reduced NF-κB activation seen in response to *Shigella* mutants lacking these effectors.

Independent of its role in cell invasion, OspB was able to activate GEF-H1 dependent NF-κB activation. OspB is a MxiE regulated gene and is therefore likely expressed after *S. flexneri* invades host cells. It was therefore surprising that we recovered a reduced number of OspB deficient *Shigella* mutants in our gentamicin protection assays. OspB expressed in the cytoplasm could initiate GEF-H1 and NOD1 dependent NF-κB signaling after the escape of *S. flexneri* from the phagosome. It needs to be determined if NF-κB activation by this effector contributes to anti-apoptotic regulation supporting survival of the pathogen in the host cell.

VirA is delivered into the host cell cytoplasm near the site of bacterial entry and induces local microtubule degradation [36]. Degradation of microtubules by the VirA related effector EspG from enteropathogenic *E. coli* results in the release of various microtubule associated proteins, including GEF-H1 [9]. VirA activity is assumed to contribute to ruffle formation during *Shigella* invasion through cross talk between RhoA and Rac-1. In our studies, VirA induced responses resulted in rapid induction of cell death in epithelial cell lines tested and failed to induce NF-κB. The function of VirA might be more closely associated with the ability of *Shigella* to move within the cell than with direct cell invasion. During multiplication within the epithelium, *Shigella* secretes additional effectors including OspF and OspG. Consistent with previous findings, OspG and OspF both failed to induce NF-κB. OspG has been demonstrated to interfere with the IκBα degradation resulting in repression of NF-κB activation and downregulated inflammatory response to infection [37]. OspF has a specific phosphatase activity that dephosphorylates and inactivates MAPK leading to blockage of phosphorylation of histone H3 which is required for transcription of a subset of NF-κB regulated genes [38],[39].

In polarized epithelial cells, GEF-H1 is associated with apical polarization complexes concentrated at TJs from which it is released and redistributed to bacterial invasion sites during *Shigella* infection. Our experiments demonstrate that, in epithelial cells, GEF-H1 is essential for RhoA activation required for cell invasion by *Shigella*. Like Cdc42 and Rac, RhoA activity is required for *Shigella* entry [40]–[42]. RhoA is not critical for *Shigella* induced actin polymerization, but is required for the recruitment of ezrin to bacterial entry sites [43].

We demonstrate that RhoA activation through GEF-H1 can contribute to the activation of NF-κB upon cell invasion by *Shigella*. Invasion by extracellular and intracellular pathogens is sensed by various signaling pathways that converge to activate NF-κB which can initiate inflammatory mediator secretion, but is also a critical cell survival signal [44]. Members of the Rho family of GTPases are involved specifically in the regulation of NF-κB dependent transcription. RhoA mediated activation of atypical protein kinase C can induce phosphorylation of p65/Rel-A at serine 811, a site that is crucial for modulating the interaction between Rel-A and CREB binding protein [45]. A recent report demonstrated that Rac1 can interact and co-localize with NOD2, raising the possibility that regulators of Rho GTPases contribute specifically to intracellular innate immune recognition [46].

Rho GTPases have been shown to be important factors in TLR2, TLR4 and TLR9 signaling [45], [47]–[50]. LPS released by *Shigella* as they escape from the phagosome into the macrophage cytoplasm activates caspase-1 which induces production of IL-1β and cell death [30]. However, TLR mediated recognition was not responsible for the rapid activation of NF-κB in response to *S. flexneri*, its effectors and GEF-H1, since our experiments were carried out in HEK293 cells, which express low levels of TLR1-9 mRNA and consequently do not respond to TLR ligands [51]. This may resemble the situation encountered by *Shigella in vivo*, where intestinal epithelial cells lack membrane expression of CD14 and remain relatively refractory to activation by extracellular LPS or noninvasive Gram negative bacteria [52]–[54]. However, our results do not exclude the possible involvement of GEF-H1 or other Rho-GEFs in bacterial recognition through TLRs in other cell types or in the recognition of other pathogens.

**Figure 7:**
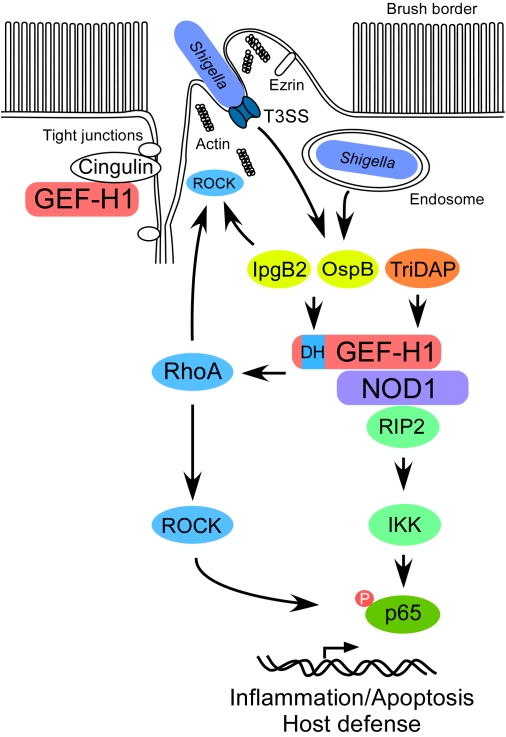
Proposed model of GEF-H1 function in the activation of innate immune responses to *Shigella* cell invasion. Upon cell attachment *Shigella* releases effectors through the T3SS into the host cell to regulate the actin cytoskeleton and facilitate invasion. This releases GEF-H1 from its binding partners in tight junctions inducing its interaction with NOD1. GEF-H1 has at least two functions in the activation of cellular defenses to microbial effectors. One mediates the sensing of muramyl dipeptides through NOD1 that is independent of its GEF activity, and the other is required for the activation of NF-κB by *Shigella* effector proteins, which requires its GEF activity mediated by the DBL homolgous domain (DH) and the activation of RhoA. RhoA activation by GEF-H1 is required for *Shigella* cell invasion through the recruitment of ezrin and regulation of the cytoskeleton potential cooperating with the RhoA mimicry of IpgB2. RhoA activation by GEF-H1 contributes to NF-κB activation through ROCK dependent phosphorylation of NF-κB proteins. Through interaction with GEF-H1, NOD1 can function in sensing the intracellular action of *S. flexneri* peptide effectors in addition to its role in the detection of peptidoglycan fragments.

Collectively, our findings support a model in which GEF-H1 is a new essential signal mediator in the activation of host defense initiated by the intracellular recognition of enteropathogens by NOD1 (**Figure 7**). GEF-H1 has two important functions, one in sensing muramyl dipeptides through NOD1 that is independent of its GEF activity, and another in the activation of NF-κB by *Shigella* effector proteins, which requires its GEF activity and the activation of RhoA. GEF-H1 and NOD1 are central components in RhoA and RIP2 dependent NF-κB signaling pathways activated upon *Shigella* cell invasion. *Shigella* release of LPS and PGN into host cells has been considered to be the main cause of the strong inflammatory response that is induced during *Shigella* infections [14]. Our experiments extend this model by demonstrating that NOD1 function is not limited to the detection of PGN fragments but, together with GEF-H1, functions in sensing the intracellular action of *S. flexneri* peptide effectors. This mechanism may allow intestinal epithelial cells to rapidly detect *Shigella* effectors and activate the NF-κB pathway to initiate survival and inflammatory signals. Our findings raise the interesting possibility that other NLR family proteins are also involved in the sensing of modifications to cellular functions by microbial effectors in addition to their role as pattern recognition receptors. Recognition of altered cell function by bacterial effectors may be important for the ability to distinguish between pathogenic and commensal microorganisms which cannot be achieved based on extracellular pattern recognition receptors alone, since many of their ligands are commonly expressed by commensal, as well as infectious microbiota.

## Materials and Methods

### Expression vectors

Plasmid encoding vsv-tagged *Canis familialis* GEF-H1 has been described previously [7]. The GEF-H1 (Y395A) mutant was generated by introducing a tyrosine to alanine mutation into residue 395 in the conserved QRITKY sequence of the DH domain that is responsible for GEF activity. Expression plasmids for dominant negative mutants of RhoA (T19N) or constitutive active mutants of Rac1 (G12V) and Cdc42 (G12V) were purchased from UMR cDNA Resource Center (University of Missouri Rolla, Rolla, MO). The pEAK13 vectors expressing the kinase deficient mutant IKKβ (K44M) or the dominant negative mutant IKKβ SS/AA in which the serines at positions 177 and 181 in the activation loop were replaced by alanines [55] were kindly provided by Dr. Ramnik Xavier, Massachusetts General Hospital, Boston, MA, USA. The pCI-HA-CARD4 vector has been described previously [56]. GFP *Shigella* effector expression vectors for IpgB1, IpgB2, OspB, OspG, OspF, IpgD and VirA were generated by RT-PCR and subcloned into pEGFP-C1 (Clontech Laboratories, Mountain View, CA).

### Antibodies and reagents

Mouse anti-GEF-H1 antibody has been described previously [57] and was kindly provided by Dr. Karl Matter, University College London, London, UK. Rabbit anti-HA (Y-11), rabbit anti-GFP (FL), mouse anti-RhoA (26C4) and goat anti-actin (I-19) antibodies were obtained from Santa Cruz Biotechnology, Inc. (Santa Cruz, CA). Mouse anti-vsv antibody (P5D4) was purchased from Sigma (St. Louis, MO). Rabbit anti-cingulin was purchased from Zymed (San Francisco, CA). Mouse anti-Rac1, mouse anti-Cdc42 and mouse anti-E-cadherin antibodies were purchased from BD Bioscience (San Jose, CA). Mouse anti-HA antibody was purchased from Roche (Indianapolis, IN). All horseradish peroxidase (HRP)-conjugated antibodies against mouse, rabbit or goat IgG were purchased from GE Healthcare (Piscataway, NJ). FITC conjugated anti-rabbit IgG, Texas red conjugated anti-mouse and anti-rabbit IgG and Cy5 conjugated anti-rabbit IgG were purchased from Jackson ImmunoResearch Laboratory (West Grove, PA). γTriDAP and αTriDAP were purchased from AnaSpec (San Jose, CA). Y27632 was purchased from Sigma.

### Cell culture and transfection protocols

HEK293 cells, MDCK cells were purchased from American Type Culture Collection (Manassas, VA) and maintained in Dulbecco's modified Eagle's medium (DMEM) with 1 g/l glucose and sodium pyruvate (Cellgro, Herndon, VA) supplemented with 10% heat inactivated fetal bovine serum and a 0.5% penicillin G/streptomycin mixture. HEK293 cells were plated 24 hours before transfection with FuGene6 (Roche Diagnostics, Basel, Switzerland) or Lipofectamine 2000 (Invitrogen, Carlsbad, CA) according to the manufacturer's protocols. MDCK cells were seeded at high density onto 0.4 µm-pore Transwell™ filter culture inserts (Corning Incorporated, Corning, NY) and studied 72–96 hours thereafter, when a transepithelial resistance of ≥600 ohm/cm^2^ was reached. To induce overexpression of genes in epithelial monolayers, MDCK cells were transfected with Nucleofector kits (Amaxa Biosystems, Gaithersburg, MD) according to the manufacturer's protocol.

### Immunofluorescence staining

Epithelial monolayers were washed three times with HBSS buffer containing Mg2^+^ and Ca2^+^, pH 7.4, (HBSS^+^) and fixed with acetone for 30 seconds at −20°C or with methanol for 20 minutes at −20°C. For blocking, fixed monolayers were incubated with 5% normal donkey serum for 30 minutes at room temperature. Primary antibodies diluted with 2.5% normal donkey serum were incubated for 16 hours at 4°C. Dilution ratios were 1∶50 for anti-GEF-H1, 1∶100 000 for anti-vsv, 1∶200 for anti-cingulin and anti-E-cadherin antibodies and 1∶500 for anti-HA antibody. After washing three times, samples were incubated with secondary antibodies (1∶500 for each secondary antibody).

### Protein separation and immunoprecipitation

Cells prepared as described above were washed with HBSS^+^ and proteins separated into Triton X-100-soluble and -insoluble fractions or homogenated in NP-40 buffer (1% NP-40, 20 mM Tris-HCl at pH 7.4, 150 mM NaCl, 2 mM EDTA, 2 mM EGTA, 4 mM Na_3_VO_4_, 40 mM NaF). Electrophoresis and transfer were performed as previously described [11]. Membranes were blocked with 3% non-fat dry milk in Tris-buffered saline (TBS) at room temperature for 1 hour and incubated with primary antibodies diluted in blocking solution to a ratio of 1∶1000 at 4°C overnight. After washing in TBS with 0.05% Tween-20 (TBS-T), membranes were incubated with appropriate horseradish peroxidase conjugated secondary antibody diluted in blocking buffer for 1 hour at room temperature. Blots were washed 3 times with TBS-T and hybridized bands were detected by Amersham ECL Western blotting detection reagent (GE Healthcare, Piscataway, NJ). HEK293 cells were transfected in 6 well plates using Fugene6 transfection reagent (Roche) according to the manufacturer's protocol. After 48 hours, proteins were separated as described above. Lysates were incubated with protein G plus agarose (Calbiochem, San Diego, CA) at 4°C for 30 minutes and pre-cleared. Pre-cleared lysates were incubated with anti-vsv or anti-HA antibodies at 4°C overnight followed by incubation with agarose beads at 4°C for 4 hours. Precipitated proteins were collected by centrifugation and washed 3 times in washing buffer (0.5% NP-40, 20 mM Tris-HCl at pH 7.4, 150 mM NaCl, 2 mM EDTA, 2 mM EGTA, 4 mM Na_3_VO_4_, 40 mM NaF). After washing, proteins were boiled with SDS-PAGE sample buffer at 95°C for 10 minutes for elution and detected by Western blotting as described above.

### GTPase activation assay

The quantification of cellular activated small GTPases was performed by precipitation with a fusion protein consisting of GST and the Rho binding domain of Rhotekin (GST-RBD) or the Rac binding domain of PAK1 (GST-PBD). Briefly, HEK293 cells were lysed in ice-cold cell lysis buffer (1% NP-40, 50 mM Tris-HCl at pH 7.4, 150 mM NaCl, 10 mM MgCl_2_, 5% glycerol and 1 tab of Complete Protease Inhibitor Cocktail Tablets) (Roche) and cleared by centrifugation at 12 000 g at 4°C for 10 minutes. Cleared lysates were incubated with GST-RBD or GST-PBD bound beads (20 µg) at 4°C for 1 hour. After washing three times, GTP bound small GTPases were captured onto beads and total small GTPases in cell lysates were detected by Western blotting using monoclonal anti-RhoA, Rac1 or Cdc42 antibodies. GTP bound small GTPase amounts were normalized to the total amount of small GTPases in cell lysates by densitometry analysis.

### Dual-luciferase assay

Luciferase assays were performed 24 hours after transfection of different vectors using Dual-Luciferase Reporter Assay System (Promega, Madison, WI) according to the manufacturer's instructions. *Renilla* luciferase activity was used as an internal control. HEK293 cells were transfected with 0.01 µg of pNF-κB-luciferase Firefly (Clontech), or 0.05 µg pGL3b-IL8 reporter construct containing 468 bp of human IL-8 promoter, and 0.001 µg of pRL-0 vector (Promega) and 0.15 µg of different vectors with FuGene6 transfection reagent or Lipofectamine 2000 as described above. For control experiments, empty vectors of indicated expression vectors were utilized. All experiments were carried out at least 3 times.

### Small interfering RNA (siRNA) mediated inhibition of gene expression

Both NOD1 siRNA, GEF-H1 siRNA and RIP2 siRNA were purchased from Santa Cruz Biotechnology. HEK293 cells were plated 24 hours before transfection in 24 well plates and then transfected with 0.01 µg of NF-κB reporter construct and 0.001 µg of pRL-0 vector and, depending on the experiments, also with 0.15 µg of GFP *Shigella* effector expression vectors (IpgB1, IpgB2, OspB, or control vector) in the absence or presence of NOD1 siRNA or GEF-H1 siRNA (40 nM). Cells were incubated for 72 hours and stimulated with γTriDAP or control (0.5 µg/mL) for 24 hours.

### Real Time PCR

Total RNA from MDCK cells was isolated using TRIzol reagent (Invitrogen) according to the manufacturer's protocol. The cDNA templates were synthesized using the iScriptTM cDNA synthesis kit and quantitative PCR reactions were performed with iQTM SYBR green PCR supermix (both from Bio-Rad, Hercules, CA). Quantitative RT-PCR analysis was performed using the Bio-Rad iQ5 System and gene expression levels for each individual sample were normalized to GAPDH. The primers used were the following: canine GEF-H1 sense 5′- GACTTTGCAGCCGACTCATGG-3′, antisense 5′- TCCTGGCGCTCCTCGTCGG-3′ and canine GAPDH sense 5′- TGTCCCCACCCCCAATGT-3′, antisense 5′- ACCCGGTTGCTGTAGCCA.

### *S. flexneri* growth condition and stimulation of epithelial cell monolayers

A wild type strain of *S. flexneri* serotype 2a (2457T) was grown at 37°C in trypticase soy broth (TSB). One hundred microliters of a stationary phase culture liquid after overnight culture was used to inoculate 10 ml of TSB and bacteria were grown in a shaking incubator for 2 hours at 37°C to mid-exponential phase as described previously [11]. Monolayers on the permeable filter supports were serum starved overnight and gently washed with HBSS^+^ three times. Bacteria were administered to the monolayers from the apical side in a MOI of 200. Cells were incubated at 37°C for the time periods indicated subsequent to 10 minute centrifugation at 1600 rpm at room temperature. After extensive washing with HBSS^+^, cells were fixed for immunolabelling or lysed for protein separation or NF-κB activation assays. The same protocol was used for HEK293 cells plated on 24-well plates. Generation of GFP-*Shigella* was based on the strategy described previously [58].

### Gentamicin protection assay

Gentamicin protection assay was performed as described previously. Briefly, bacterial samples at a MOI of 200 were administered to monolayers and incubated for 90 minutes at 37°C. After washing three times, cells were treated with 480 µg/ml gentamicin for 60 minutes at 37°C. Intracellular bacteria were released by lysis with 1% Triton X-100 solution after washing 5 times. Cell lysates were sequentially diluted and plated on LB agar plates for counting of colony forming units. The same protocol was used for HEK293 cells plated on 24-well plates. All experiments were carried out at least 3 times and representative data are included.

### Statistical analysis

All results are expressed as the mean±SD. Statistical analyses were performed using a statistical software package, Statview 4.5 (Abacus Concepts, Berkeley, CA). Differences among samples were assessed using a Student's *t*-test, and *p* values of less than 0.05 were considered statistically significant.
